# Impact of quantization on various CNN architectures for bone fracture detection

**DOI:** 10.3389/fmedt.2026.1892595

**Published:** 2026-08-12

**Authors:** Sushil Krishnan, Yokesh Babu Sundaresan

**Affiliations:** School of Computer Science and Engineering, Vellore Institute of Technology, Vellore, Tamil Nadu, India

**Keywords:** bone fracture, deep learning, edge computing, image classification, medical image analysis, quantization, smart healthcare

## Abstract

The skeletal system, comprising bones, plays a crucial role in physical support and locomotion. Any deformation of bones, due to trauma or medical conditions like osteoporosis, can lead to a bone fracture. Bone fractures are painful conditions requiring immediate medical attention. Automating fracture detection can play a significant role in early diagnosis and intervention for bone fractures. Convolutional neural networks (CNN) have shown promising results in multiple medical imaging applications. There is a wide variety of convolutional neural network architectures. Due to their high computational and power requirements, they are not being adopted in resource-constrained environments. Quantization is a technique by which the size and power consumption a model are minimized with minimal loss of accuracy. It is performed by converting higher-precision data types to lower-precision data types. It enables the adoption of complex artificial intelligence models in resource-constrained environments such as mobile devices, edge devices, and IoT devices. The work focuses on finding the optimal convolutional neural network architecture and quantization method for bone fracture detection. For comparison, seven models were selected from different convolutional neural network architectures, namely, LeNet, AlexNet, VGG 19, Inception-V3, ResNet-152, MobileNetV2, and EfficientNet-B0. A baseline transformer, ViT-B/16, was also considered. Dynamic range quantization, float-16, and int-8 quantization were performed to analyze their impact on the diagnostic and deployment performances of the model. TOPSIS and Cohen's *d* analyses were performed. It was observed that lightweight models, such as LeNet and MobileNetV2, benefit greatly from quantization. This study serves as a practical guide for optimizing in a resource-constrained environment.

## 1 Introduction

Bones constitute a vital part of the human body and play a significant role in physiology and structural balance. The human body consists of 206 bones. Any partial or complete deformation of bone is a medical condition called a bone fracture (1). It usually occurs when force is exerted beyond the level that the bones can withstand. The presence of certain medical conditions like osteoporosis and bone cancer can also increase the susceptibility to bone fracture. Fractures can be localized to a single point or occur in multiple places, resulting in bone fragmentation. Globally, millions of cases are observed every year due to accidental or intentional causes. While most fractures can be diagnosed from a plain X-ray, some fractures are not visible on it. In such cases, other imaging tests, such as bone scintigraphy, computed tomography, or magnetic resonance imaging, are required (2).

Bone fractures require timely diagnosis based on radiological analysis, followed by appropriate interventions such as cast and surgeries. Prompt medical attention plays a significant role in minimizing the risk of infection and the length of hospital stay (3). In terms of diagnosis, radiological imaging and its interpretation are primary bottlenecks. Challenges faced include the unavailability of expert radiologists, poor imaging quality, subtle fractures, diagnostic fatigue, and resource constraints. Automating bone fracture detection across various image modalities using artificial intelligence can greatly improve the timeliness of medical care. These systems can handle a huge diversity of bones in the skeletal system (4). Artificial intelligence refers to a set of techniques that enable machines or computers to perform diverse tasks. They are proven to assist the medical field immensely; particularly, machine learning and deep learning can accelerate medical image analysis (5). These techniques can be applied across the workflow at various stages, including image enhancement, classification, localization, and decision support.

Image classification models are used to identify radiological images that contain fractures. They can help health care institutions overcome the stated limitations. Transfer learning in deep learning enables machines to leverage knowledge gained from solving one problem to solve a different one. This method has led to a large number of pretrained models that can be retrained on smaller datasets and achieve greater accuracy (6). This method can address the issues of diversity in bones, as knowledge gained in one domain can be transferred to solve problem in another domain. The large and complex nature of deep learning models makes them resource-intensive, thereby hindering their adoption in low-resource settings. To effectively address the issues related to resource constraints, various compression methods such as quantization, pruning, low-rank approximation, and knowledge distillation are used. Quantization reduces model size by approximating numerical components with lower-precision data types (7). While quantization improves the efficiency of the model, the impact of various quantization methods on different model architectures varies significantly. An end-to-end experimental study is required to quantify them and provide insights for developing practical solutions. By making the models robust and resource-efficient, effective wide-scale adoption to improve health services is possible.

Our main contributions to the work are listed as follows:

- We trained a model from various classes of convolutional neural networks for bone fracture detection and compared their performances. In addition, a model from modern transformer architecture was also considered for the study. LeNet, AlexNet, VGG-19, Inception-V3, ResNet-152, MobileNetV2, EfficientNet-B0, and ViT-B/16 were trained and evaluated.
- All trained models were quantized and evaluated using dynamic range quantization, float-16 quantization, and int-8 quantization.
- The performance of non-quantized and quantized models was compared in terms of diagnostic performance, model size, and inference latency.
- The impact of quantization on different convolutional neural network architectures was experimentally quantified.

This article is organized as follows: Introduction section provides an overview of bone fractures, limitations in fracture diagnosis, deep learning, and quantization; Literature Survey section presents the existing relevant research works; Methodology section provides clear insights on research work carried out; Results and Discussion section highlights the performance metrics of non-quantized and quantized models and compares them; Conclusion section presents an impact of quantization in bone fracture detection; and finally, Future work and references are presented.

## 2 Literature survey

Medical fields greatly benefited from image processing techniques; notable examples include wavelet transformations for biomedical image compression and a scale-invariant feature transformation algorithm for feature extraction from biomedical images. A scale-invariant feature transformation algorithm combined with backpropagation neural networks was able to accurately detect X-ray radiographs with bone fractures (8). There are 14 types of learning in artificial intelligence, each with its own pros and cons. Based on the scenario, multiple learning methods can be ensembled to perform a specific medical image analysis task. These models can be used to perform methodological tasks (detection, localization, segmentation, registration, and classification) or clinical tasks (image-guided intervention, computer-aided diagnosis, biometric measurement, and therapy) or a particular anatomical application, such as the eye, brain, abdomen, and heart (9).

The huge volume, veracity, and velocity of data, along with improved computational hardware, have motivated researchers to develop various deep learning models that can perform segmentation and classification. Deep learning models are trained on several image modalities, including CT, MRI, fundus, pathological, ultrasound, X-ray, and endoscopic images (10). Image formats like DICOM and NIfTI are being developed to store three-dimensional biomedical images for convenient use in deep learning applications (11). An ensemble method with five different deep learning models was developed to accurately segment fractures in wrist X-ray images. The final wrist fracture detection combo was proposed after testing various combinations across 26 different models, and it achieved an average precision of 86.39% (12).

Over the years, various classes of deep learning architectures have been proposed, and their abilities have progressively improved. While these models varied significantly in terms of complexity and generalization, convolution remains a cornerstone of their strength. Convolution-based architectures became popular with the success of LeNet-5, which used layered convolutions for feature extraction (13). A pivotal moment in the 2012 ImageNet Challenge was the introduction of AlexNet, which was well recognized for its superior performance due to architectural improvements and the use of GPUs instead of CPUs, setting the tone for future developments (14). To enable the model to learn a rich data representation and achieve higher performance, models were made complex, computationally intensive, and pretrained. This era of transfer learning produced architectures such as VGG, ResNet, and Inception (15). While performances plateaued, the need for optimal models in terms of resources was required to cater to the needs of edge computing (16) and the wide-scale adoption of artificial intelligence. A comparatively minimal computational and memory footprint was achieved through depthwise separable convolutions in MobileNet and compound scaling in EfficientNet (17). Recently, convolutional network's constraints have been bypassed through the self-attention mechanism of transformers to capture more context in models like Vision Transformer (18). All these architectural developments, from heavy classical networks to attention-based transformers, have led to the reliable adoption of AI across healthcare, from hospital management to disease diagnosis (19). Radiological imaging is extensively used in bone health diagnosis, making it a natural application domain for the discussed models. Hybrid approaches featuring ensemble models and other techniques, such as fuzzy methods, are used for osteoporosis detection, a precursor to fracture (20). These approaches enable the exploitation of the benefits of multiple architectures.

Explainability is seen as a crucial factor in medical applications to avoid mishaps. GoogleNet and transfer learning are found to be efficient in detecting fractures in pelvic X-ray images, achieving an accuracy of 98.5%. The same configuration has been used to develop an explainable artificial intelligence framework for pelvic fracture detection (21). Deep learning radiomic features, extracted via transfer learning and hand-crafted features, can be combined to generate radiomic signatures. Machine learning models trained on these signatures were used to differentiate acute and chronic vertebral compression fractures (22).

Deep neural networks have consistently outperformed human experts across multiple tasks. The limitations preventing their adoption include larger model size and huge computational hardware requirements. To overcome this challenge, compression techniques such as weight quantization and pruning can be applied. These methods effectively reduce their size and decrease the computational requirements (23). This can enable the deployment of sophisticated models on resource-constrained edge devices. Quantization of convolutional neural networks for various tasks has been shown to improve accuracy and reduce memory requirements by factors of 3.5–6.4 times (24). Adaptive quantization and radical residual connection can be applied to achieve 14× speedup and 15× compression. The same has been tested on the LiTS and BRATS2020 datasets for the medical image segmentation task (25).

The Internet of Medical Things (IoMT) is a type of IoT specifically designed to cover all aspects of medical applications. It is intended to serve various tasks such as identity recognition, drug delivery, vital monitoring, biomarker monitoring, and disease detection. It is powered by a combination of multiple technologies, such as sensors, actuators, cloud, edge, and artificial intelligence (26). The beauty lies in a hybrid approach that uses both cloud computing and edge computing. Tasks that require immediate response are performed in edge devices with fewer resources (27). In such cases, model quantization plays a significant role in minimizing model size and resource requirements. An IoMT-based system has been developed to detect osteosarcoma, a type of bone tumor. The system detects the condition from whole slide images with an accuracy of 99.3%, and the study also highlights the significance of edge and fog computing to reduce the load on centralized servers (28).

Extensive work exists on the applications of deep learning and other artificial intelligence techniques in health care and bone fracture detection. Fewer works focus on providing an end-to-end comparison of various approaches for practical solutions. This study addresses these gaps by offering broad insights into various architectures and quantization techniques to support the deployment of efficient models in resource-constrained environments.

## 3 Methodology

**Figure 1:**
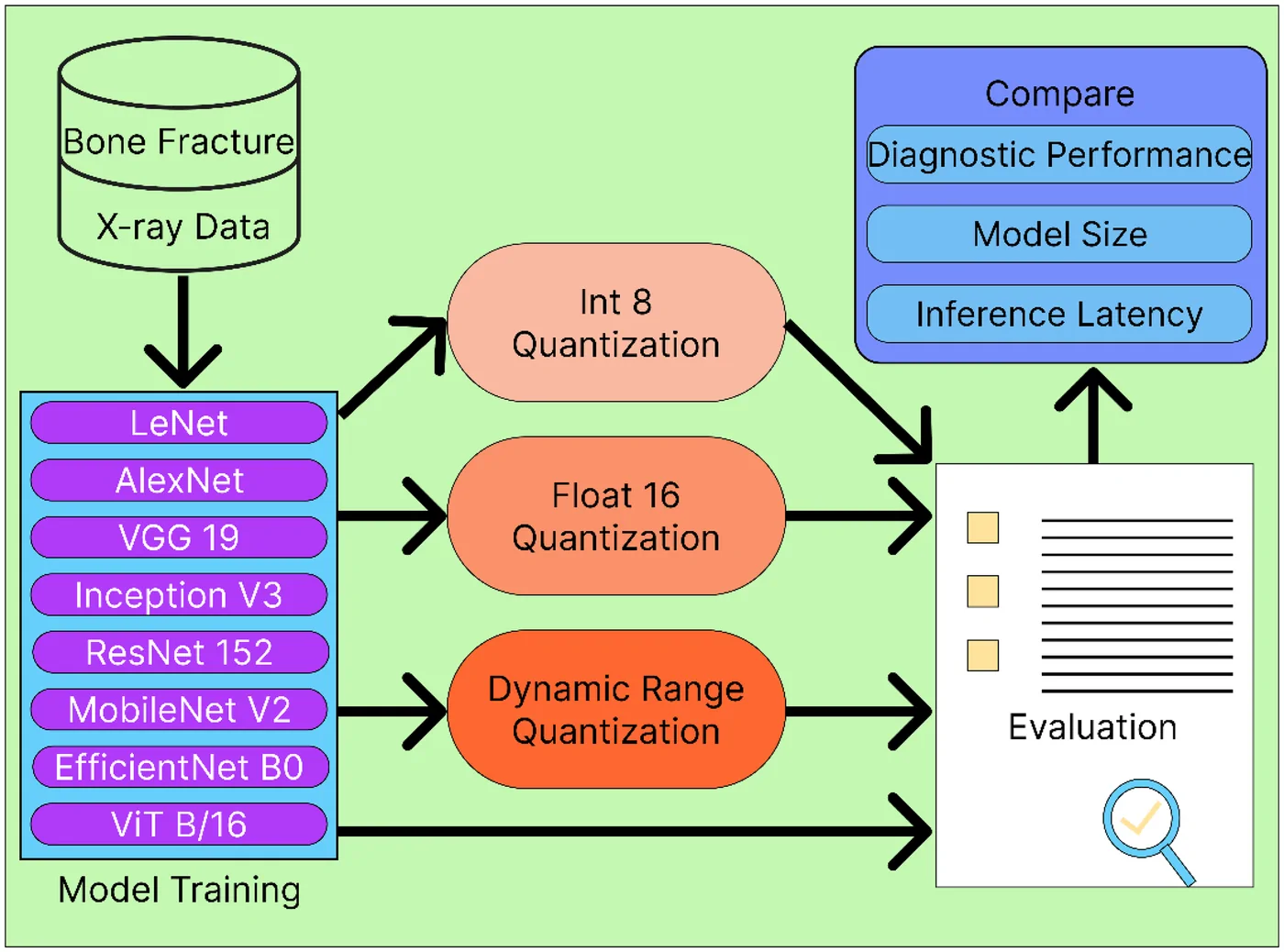
Workflow of the study evaluating the impact of quantization on various models for bone fracture detection.

The study focuses on identifying the impact of quantization on various convolutional neural networks (CNN) architectures in bone fracture detection. The workflow architecture in Figure 1 provides a clear overview of the work carried out. The multiregion bone fracture X-ray image dataset was retrieved from the Kaggle data repository (29). The dataset comprised 10,581 radiographic images from all anatomical regions. A deep learning model was chosen from each class of convolutional neural network architecture, from the oldest LeNet to the latest EfficientNet, and a transformer model was also considered. The models chosen were LeNet, AlexNet, VGG-19, Inception-V3, ResNet-152, MobileNetV2, EfficientNet-B0, and ViT-B/16. Following a thorough data preprocessing, all these models were trained. All trained models were quantized using dynamic range quantization, float-16 quantization, and int-8 quantization separately. All trained non-quantized models, dynamic-range-quantized models, float-16-quantized models, and int-8-quantized models were evaluated for performance, size, and latency.

### 3.1 Data preprocessing

**Figure 2:**
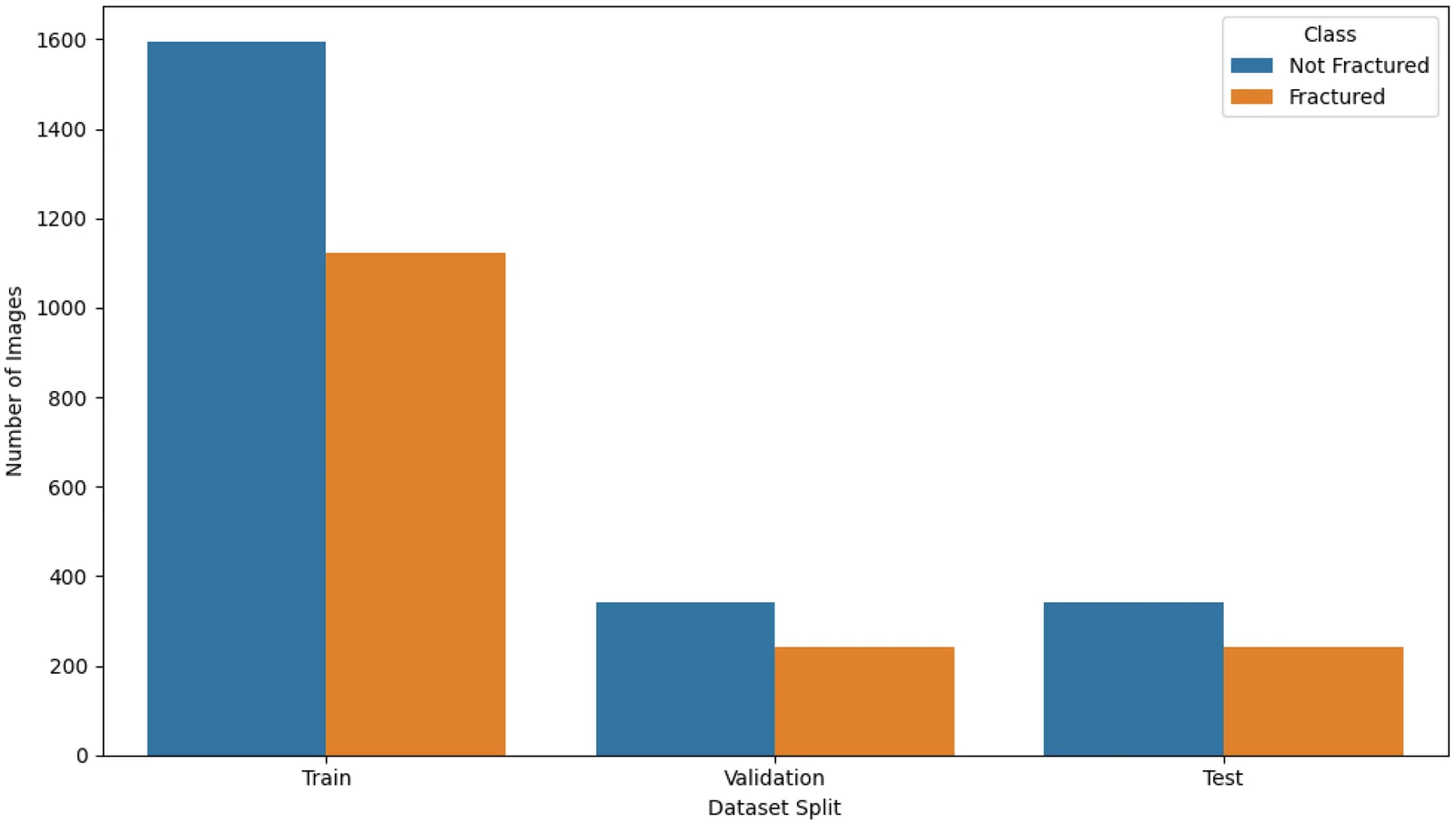
Class distribution of training, validation, and test sets.

The multiregion bone fracture X-ray dataset was obtained from Kaggle, and comprehensive preprocessing was applied to ensure a reliable study. While the dataset included 10,581 radiograph images, duplications and size variations were observed to cause data leakage. MD5 hash-based duplicate detection was used to eliminate duplicates, bringing the total number of images to 3,883. The images were then resized to 224 × 224 × 3 pixels, normalized to [0,1], and stratified sampling was employed to maintain class distribution during splitting. The cleaned dataset was split into 70% training, 15% validation, and 15% test sets. Figure 2 shows the class distribution of the splits.

### 3.2 LeNet

LeNet is one of the earliest convolutional neural networks, originally developed for recognizing simple digits in images (30). It was selected as a lightweight baseline due to its shallow architecture and lower complexity. The architecture has seven layers comprising two convolution layers, two average pooling layers, two dense layers, and a sigmoid output neuron. It was inspired by the original LeNet model proposed in 1998; a few modifications were made to make it better suited to our application. The input size was increased from (32 × 32) grayscale to (244 × 244) RGB to capture more anatomical details. Convolution kernels of size 5 × 5 with stride 1 and tanh activation were used.

### 3.3 AlexNet

AlexNet is an eight-layer convolutional neural network. It consists of five convolution layers and three dense layers (31). It was selected because it was the first GPU-based large-scale deep learning architecture proven to be effective for image classification. Its significantly larger representational abilities than LeNet can be attributed to large layers and deep feature extraction. Kernels of size 11 × 11, 5 × 5, and 3 × 3 were utilized along with ReLU activation. While the first layer uses a stride of 4, subsequent layers use the default stride of 1. Dropout layers with a dropout rate of 0.5 were implemented to reduce overfitting and improve generalization.

### 3.4 VGG-19

VGG-19 is a convolutional neural network with 19 layers. It is pretrained with millions of images from the ImageNet repository. It was selected because it is a deep sequential model with small convolution kernels for progressive feature extraction. It comprises five blocks, each consisting of a few convolution layers followed by a max pooling layer (32). It uses 3 × 3 convolution filters uniformly and ReLU as an activation layer. Its simplicity and effectiveness have greatly influenced the subsequent models. While the pretrained convolutional blocks were retained, the classification head was replaced with a global average pooling layer, a 512-neuron dense layer, dropout with a rate of 0.5, and a sigmoid output layer.

### 3.5 Inception-V3

Inception-V3 is a 48-layer deep convolutional neural network. It is pretrained with millions of images from the ImageNet repository. It was selected because it has a multiscale feature extraction. It has several optimizations and is more extensive than its previous versions (33). It uses auxiliary classifiers as regularizes. Major optimizations include factorization into smaller convolutions, the use of auxiliary classifiers, spatial factorization, and efficient grid size reduction. Factorized convolutions are used with ReLU activation. Top classification layers were removed, and global average pooling, dropout, and a sigmoid output layer were added to train it as per the dataset.

### 3.6 ResNet-152

ResNet-152 is a deep convolutional neural network with 152 layers (34). The number of hidden layers offers an advantage for feature extraction; however, there is also a vanishing gradient and degradation with increasing numbers of hidden layers. This problem is efficiently handled by having shortcut connections in residual neural networks. It also speeds up the training process. It was also pretrained on the ImageNet repository. It was selected because it is a very deep residual network. It consists of residual blocks with 3 × 3 convolutions and ReLU activation. Top classification layers were replaced by global average pooling, a dense layer, batch normalization, dropout, and a sigmoid output layer.

### 3.7 MobileNetV2

MobileNetV2 is a convolutional neural network model with 53 layers. It has some new layers, such as the inverted residual layer and a linear bottleneck layer. It works very effectively in mobile and embedded applications. The convolutions used are depth-wise separable. It provides higher accuracy with minimal parameters and computations. It was also pretrained on the ImageNet repository (35). It was selected because it is optimized for resource-constrained environments. It uses ReLU6 activation, a pretrained backbone, global average pooling, batch normalization, dropout, and a sigmoid output layer.

### 3.8 EfficientNet-B0

EfficientNet-B0 maintains optimal complexity in terms of network depth, width, and resolution through compound scaling. It was selected because it represents modern lightweight architecture with superior performance (36). Backbone uses a squeeze-and-excitation mechanism with MBConv blocks and swish activation. As with MobileNetV2 models, the classification head was replaced in EfficientNet-B0.

### 3.9 Vit-B/16

The vision transformer approach uses self-attention and a transformer encoder in place of the convolution feature extractor of CNNs. It was selected for the analysis of modern transformers along with CNNs. In B/16, images are divided into 16 × 16 patches that are handled by 12 transformer encoder layers with 12 self-attention heads and an embedding dimension of 768 (37). The classification head is composed of global average pooling, dropout, and sigmoid output.

### 3.10 Quantization

Quantization is a pivotal technique utilized to minimize the memory requirements and computational requirements of running artificial intelligence models. It reduces model size by replacing higher-precision weights and activations with lower-precision data types, such as 8-bit integers. Reducing the size results in lower memory requirements for both storage and execution. It also reduces the complexity of mathematical operations, thereby reducing power consumption (38). The primary goal of quantization is to enable models to run in resource-constrained environments, such as edge, mobile, and IoT devices. Usually, models are represented using high-precision 32-bit floating-point numbers for weights and activations. These higher-precision numbers can be converted to lower-precision data types such as float-16, int-16, and int-8. All these processes must be carried out without a significant compromise in the performance metrics of the model. There are multiple approaches to quantization, namely, post-training dynamic quantization, post-training static quantization, and quantization-aware training (39).

### 3.11 Dynamic range quantization

Dynamic range quantization is a type of post-training quantization in which only the weights are quantized from floating-point to 8-bit integer precision. Activations are dynamically quantized based on the runtime requirements. Here, the process is simple, as the focus is only on converting the weights; in some cases, it might lead to minimal shrinkage. The outputs are still produced in a higher-precision format, leading to incompatibility with certain hardware. The models were converted to TFLite using the TFLiteConverter in the TensorFlow Lite API. No further processing was required, as the default scheme in the converter is dynamic range quantization. It identifies the range for each layer and accordingly quantizes. The models were saved for further examination.

### 3.12 Float-16 quantization

Float-16 quantization is a type of post-training static quantization. All numerical values associated with the model, including the weights and activations, are converted to the 16-bit floating-point data type. This guarantees a reduction in model size by a factor of two. Apart from size reduction, it is more advantageous, as the loss of accuracy is very minimal, and certain hardware can perform these operations very effectively. The models were converted to TFLite using the TFLiteConverter in the TensorFlow Lite API. It involves manually declaring the optimization parameters to perform float-16 quantization. It quantizes all numerical values indiscriminately in models to float-16 precision. The models were saved for further examination.

### 3.13 Int-8 quantization

Int-8 quantization is a type of post-training static quantization. Similar to float-16 quantization, all numerical values are converted to 8-bit integer precision. While, in theory, it offers the least complexity in terms of computation, size, and time, general-purpose systems suffered because they were largely optimized for floating-point operations. Consequently, it proved beneficial for architectures natively built for integer arithmetic, such as edge devices. The TensorFlow TFLite Converter was used; it requires a representation dataset for calibrating quantization ranges. Both input and output tensors were configured to use 8-bit integer precision.

### 3.14 Training parameters

The dataset used was the multiregion bone fracture X-ray dataset from Kaggle (https://www.kaggle.com/datasets/bmadushanirodrigo/fracture-multi-region-x-ray-data). To ensure fair evaluation of all architectures, a fixed random seed of 42 was used. Random horizontal flips and rotations of up to 20° were used to improve randomness and generalizability. All models were optimized for binary cross-entropy loss using the Adam optimizer and an initial learning rate of 1 × 10^−4^. Training was run for up to 50 epochs with a batch size of 32. Early stopping was based on validation loss with patience of 10 epochs, and the learning rate was optimized with the ReduceLROnPlateau scheduler (factor = 0.5, patience = 5, minimum learning rate = 1 × 10^−7^). LeNet and AlexNet were trained from scratch, and the remaining models were pretrained with ImageNet weights from TensorFlow Keras. The backbones of pretrained models were frozen, and the newly added classification head was optimized during training. The notebooks were run on Google Colaboratory. The system utilizes an Intel(R) Xeon(R) CPU @ 2.00 GHz with 12.7 GB of RAM and an NVIDIA Tesla T4 GPU with 16 GB of VRAM.

## 4 Results and discussion

### 4.1 Model training

**Figure 3:**
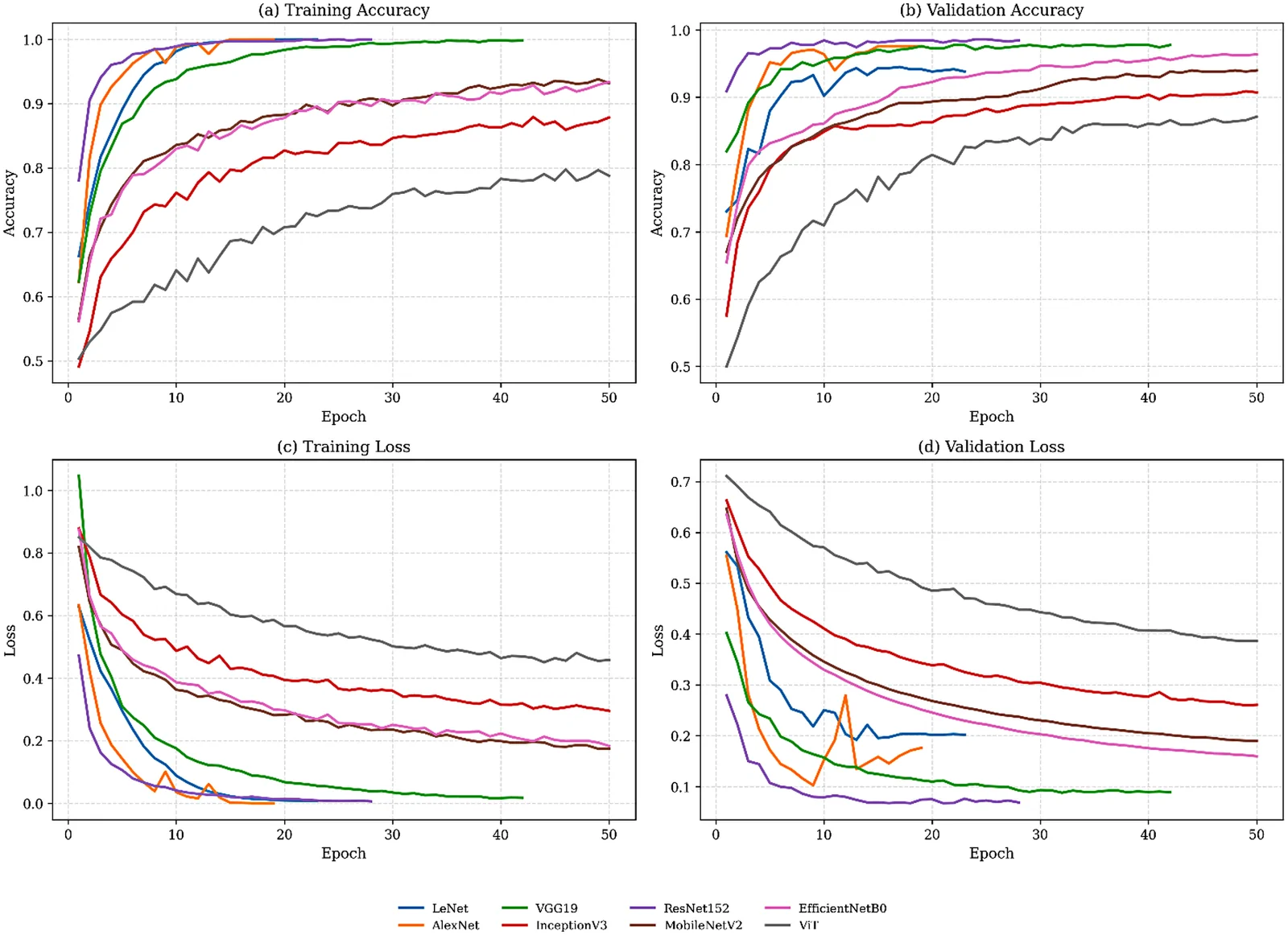
Training and validation curves of the FP32 model: (**a**) training accuracy, (**b**) validation accuracy, (**c**) training loss, and (**d**) validation loss.

All the selected models were trained on the multiregion bone fracture X-ray dataset. Training accuracy, validation accuracy, training loss, and validation loss are shown in Figures 3a–d, respectively. The learning curves indicate stable convergence for most models, with no significant instability except for AlexNet. ResNet-152 is observed to converge faster and achieve the highest training and validation performance. It is closely followed by AlexNet and LeNet. The remaining architectures showed gradual convergence, while the transformer architecture performed worse here due to its higher data requirements. Overall, the training and validation behaviors agree, which indicates minimal overfitting.

### 4.2 Comparative performance evaluation

All eight trained models were then quantized using dynamic range quantization, float-16 quantization, and int-8 quantization separately, resulting in a total of 32 models, including 8 non-quantized models (FP32) and their corresponding 8 dynamic-range-quantized models (DynamicRange), 8 float-16-quantized models (Float16), and 8 int-8-quantized models (INT8). Test accuracy, precision, recall, specificity, F1, ROC AUC, model size, and mean latency of the FP32 models are presented in Table 1. In terms of diagnostic performance, ResNet-152 performed the best, and AlexNet and VGG 19 also showed an accuracy of 97%. Lightweight architectures, such as MobileNetV2 and EfficientNet-B0, remained highly competitive despite smaller model sizes. Despite lower accuracy, LeNet showed the best latency. ViT was observed to perform poorly across all aspects. The presence of multiple aspects for practical deployment, through the evaluation of quantization and simultaneous tradeoff, is required, as carried out in this work.

**Table 1:** Performance metrics of FP32 models.

Architecture	Accuracy	Precision	Recall	Specificity	F1	ROC AUC	Model size (MB)	Mean latency (ms)
LeNet	0.928	0.942	0.880	0.962	0.910	0.949	21.41	**8**.**3**
AlexNet	0.971	0.967	0.963	0.977	0.965	0.997	222.35	165.1
VGG-19	0.973	0.967	0.967	0.977	0.967	0.995	77.40	549.5
Inception-V3	0.868	0.848	0.838	0.889	0.840	0.949	83.11	79.7
ResNet-152	**0**.**986**	**0**.**992**	**0**.**975**	**0**.**994**	**0**.**983**	**0**.**999**	225.60	314.4
MobileNetV2	0.949	0.941	0.934	0.959	0.937	0.984	**8**.**46**	10.9
EfficientNet-B0	0.964	0.962	0.950	0.974	0.956	0.991	15.30	20.4
ViT-B/16	0.858	0.853	0.792	0.903	0.821	0.918	327.45	430.7

Bold values denotes the best performance for each respective metric.

To simultaneously analyze all performance parameters and deployment parameters, a well-known multicriteria decision-making technique was used. Entropy-weighted Technique for Order Preference by Similarity to Ideal Solution (TOPSIS) analysis was performed on the results of all the models to rank them, and the results are presented in Table 2. It can be observed that lighter architectures, such as LeNet, MobileNet, and EfficientNet, topped the list and benefited the most from quantization techniques. Despite showing higher diagnostic performance, ResNet, VGG, and AlexNet were not among the top 10 due to their limitations in deployment. It can also be inferred that dynamic range quantization is better positioned than other quantization techniques, offering the best tradeoff between performance and computational requirements. It minimizes quantization-induced error by quantizing only weights and dynamically quantizing activations at inference. Lightweight architectures with fewer parameters are less sensitive and maintain performance while requiring a substantial reduction in computational needs. While int-8 quantization offers the highest compression, its inference latency can vary widely across hardware, as general-purpose processors are optimized for float operations. Edge devices supporting integer operations with special architectures will benefit from int-8 quantization. These methods enable us to tradeoff among parameters to identify the suitable model for a given scenario.

**Table 2:** Top 10 models ranked by entropy-weighted TOPSIS analysis.

Rank	Architecture	Model	TOPSIS score	Accuracy	Model size (MB)	Mean latency (ms)
1	LeNet	DynamicRange	0.707	0.928	5.36	5.3
2	LeNet	Float16	0.551	0.928	10.71	6.6
3	MobileNetV2	INT8	0.544	0.899	2.58	16.6
4	MobileNetV2	DynamicRange	0.511	0.943	2.39	27.1
5	LeNet	INT8	0.472	0.928	5.36	11.3
6	MobileNetV2	Float16	0.468	0.950	4.26	12.7
7	LeNet	FP32	0.437	0.928	21.41	8.3
8	MobileNetV2	FP32	0.398	0.949	8.46	10.9
9	EfficientNet-B0	INT8	0.379	0.796	4.68	19.9
10	EfficientNet-B0	DynamicRange	0.349	0.964	4.34	35.3

**Figure 4:**
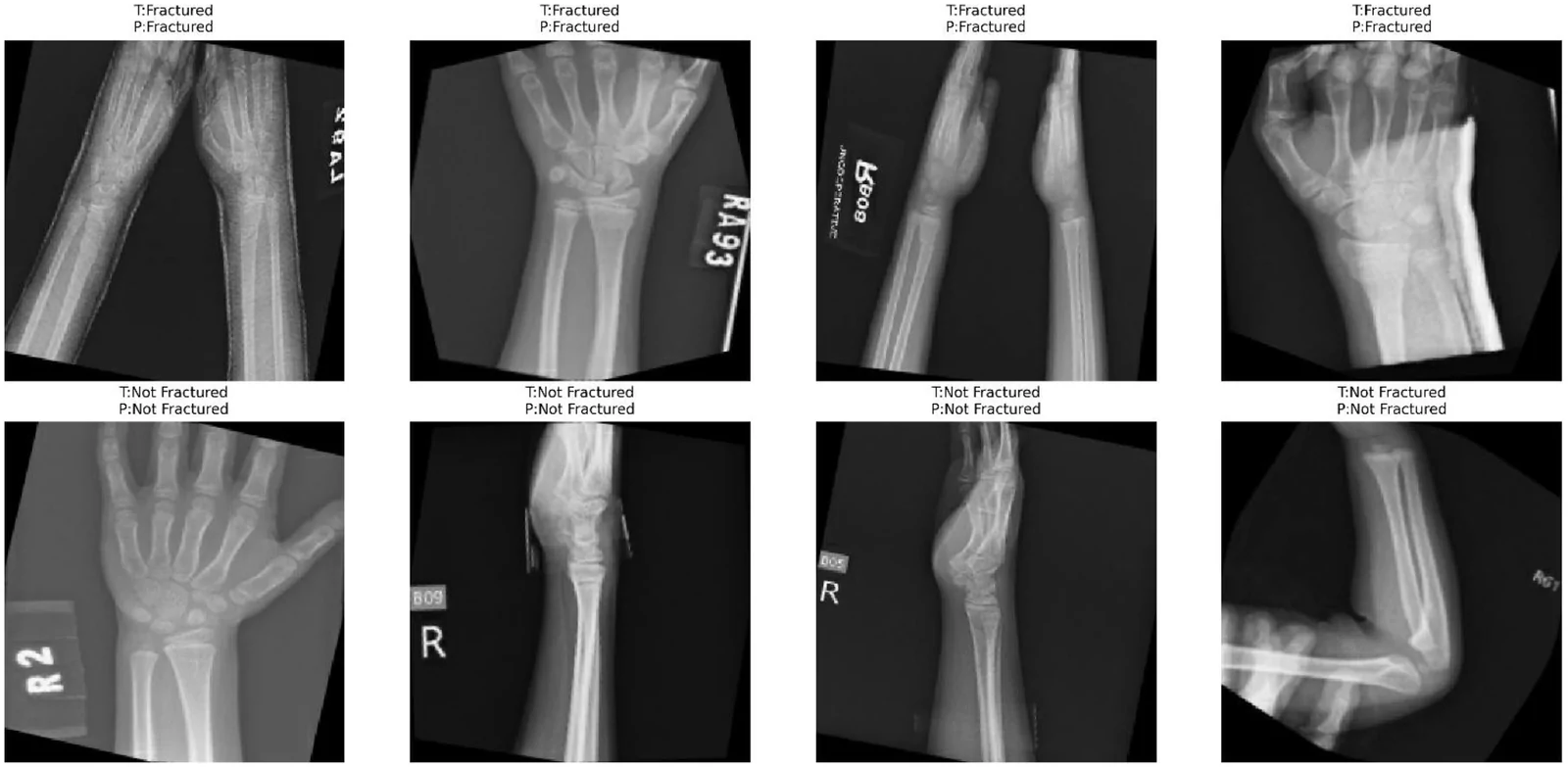
Predictions of dynamic range quantized LeNet.

Prediction examples of the top model, dynamic-range-quantized LeNet, are depicted in Figure 4.

### 4.3 Statistical analysis

To statistically demonstrate that quantization minimizes model size and inference time with minimal loss in accuracy, Cohen's *d* measure was used. It was preferred over other statistical methods, such as ANOVA, because it can quantify the magnitude of differences rather than just checking for statistically significant differences. Cohen's *d* measures the statistical difference between two groups, and its formula is given in Equation (1), where *d* stands for Cohen's *d* value, $\bar{X_{1}}$ and $\bar{X_{2}}$ are the means of the two groups being compared, and $S_{p}$ is the pooled standard deviation. Equation (2) presents the formula to calculate the pooled standard deviation, where $n_{1}$ and $n_{2}$ are the lengths of the two groups being compared and $S_{1}$ and $S_{2}$ are the standard deviations of the two groups being compared (40)

$$d=\frac{\bar{X_{1}}-\bar{X_{2}}}{S_{p}}$$ (1)

$$S_{p}=\sqrt{\frac{(n_{1}-1)s_{1}^{2}+(n_{2}-1)s_{2}^{2}}{n_{1}+n_{2}-2}}$$ (2)

**Figure 5:**
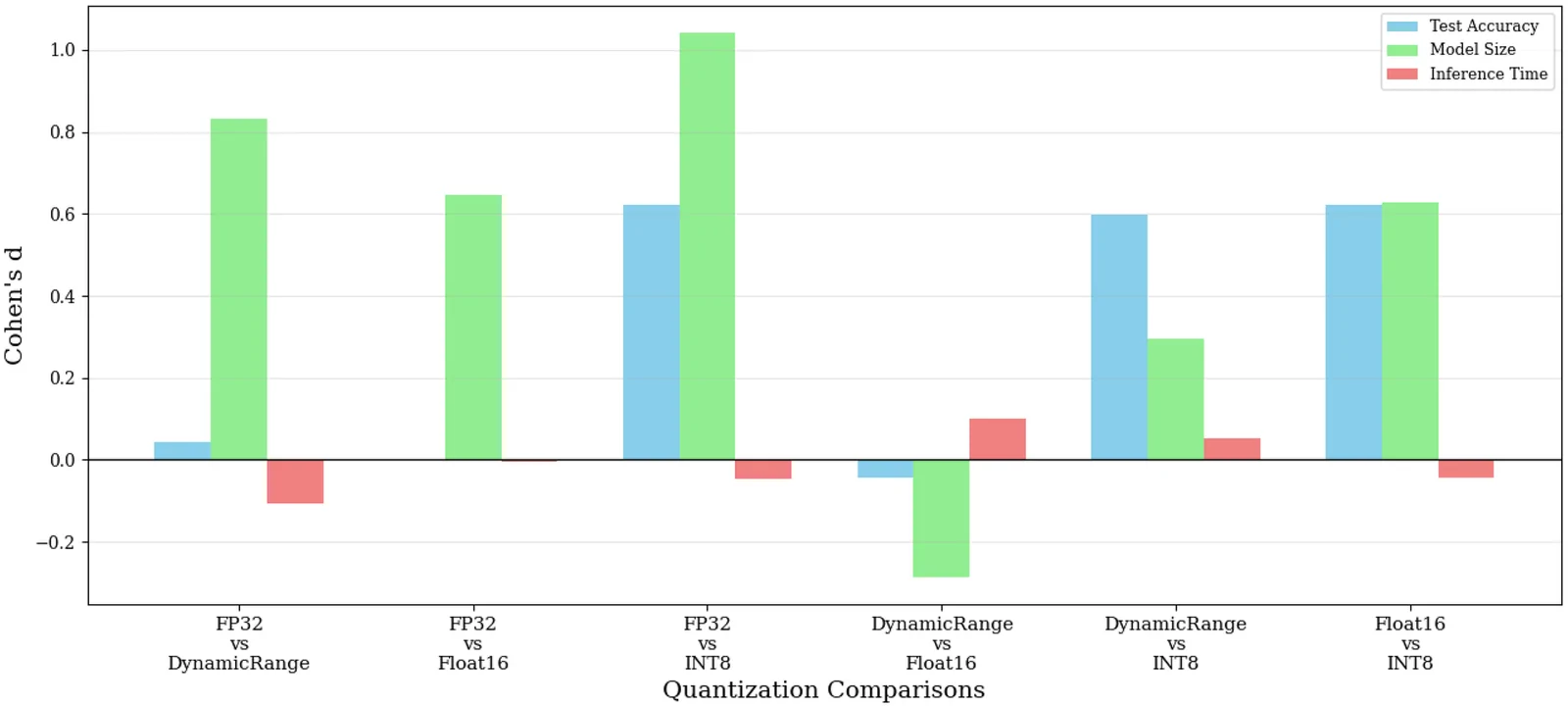
Cohen's *d* values for test accuracy, model size, and inference time.

The Cohen's *d* value was calculated pairwise for all three metrics, as visualized in Figure 5. Dynamic range and float-16 quantization showed minimal degradation in accuracy, but int-8 quantization showed greater impact on certain architectures. Model size remained significantly reduced in all quantization techniques. In the case of inference latency improvements, the observed gains are marginal relative to model size. Overall, the analysis demonstrates that post-training quantization methods substantially improve deployment efficiency with minimal performance degradation. Dynamic range quantization offers a favorable tradeoff among the three quantization techniques.

### 4.4 Discussion

Applications of convolutional neural networks in medical applications are rapidly increasing, especially their ability to process large numbers of images, which makes them a powerful tool. As highlighted in the Literature survey section, numerous studies have explored the applications of various CNN architectures for the classification of radiological images. Consistent with the literature, most of the evaluated models are also highly effective at classifying images. Deep architectures are achieving higher diagnostic performance; however, deployment aspects also play a vital role in determining the optimal model for practical deployment. Through TOPSIS analysis, architectures with lighter designs were shown to benefit more from quantization techniques. Cohen's *d* also built on the results to confirm that gains in deployment aspects are accompanied by only a minimal degradation in predictive performance. Findings show the impact of quantization across various architectures and justify the need for this elaborate study to deeply analyze and tradeoff.

## 5 Conclusion

A comprehensive analysis of the impact of quantization on various convolutional neural network architectures for bone fracture detection was performed. The work covered eight architectures spanning classical neural networks (LeNet, AlexNet), deep CNNs (VGG-19, Inception-V3, ResNet-152), lightweight networks (MobileNetV2, EfficientNet-B0), and a transformer model (ViT-B/16). All models were further individually optimized using three post-training quantization techniques: dynamic range quantization, float-16 quantization, and int-8 quantization. All models were evaluated for diagnostic performance, model size, and latency. From the results, it was found that ResNet-152 achieved the highest diagnostic performance, whereas lightweight architectures, such as MobileNetV2 and LeNet, benefited most from quantization and offered a good tradeoff between performance and deployment aspects. Dynamic-range-quantized LeNet was identified as the most favorable model using the TOPSIS analysis, and Cohen's *d* test confirmed the impacts of quantization. It was shown that quantization greatly reduces model size and marginally reduces inference latency, along with minimal degradation of accuracy.

This study demonstrates the practical usefulness of quantization techniques to improve healthcare services. The models evaluated in this study can support resource-constrained healthcare centers, mobile units, and edge devices for decision support in bone fracture screening. They can facilitate faster diagnosis and minimize dependence on cloud infrastructure, reducing costs and privacy concerns. Although this study is limited to post-training quantization and a single dataset, rigorous data preprocessing reduced the dataset size, eliminated data leakage, and ensured a realistic generalization assessment. However, larger datasets can be used to evaluate the learning abilities of a complex model during optimization. These findings leave us with a significant scope for future work in model optimization for resource-constrained environments across domains. Future work should investigate other optimizations, such as quantization-aware training, pruning, clustering, multiple datasets, and multicenter datasets, to evaluate robustness across different modalities, patient populations, and clinical settings, along with hardware-specific optimizations. Quantization can enable the adoption of artificial intelligence techniques across a wider range. It enables resource-constrained and privacy-sensitive environments to deploy such models on their edge devices. It can also play a vital role in the medical field, where timely analysis and decision-making are required.

## Data Availability

Publicly available datasets were analyzed in this study. This data can be found here: https://www.kaggle.com/datasets/bmadushanirodrigo/fracture-multi-region-x-ray-data
